# Levofloxacin-Resistant Streptococcus gordonii Infective Endocarditis in an Elderly Patient: A Case Report

**DOI:** 10.7759/cureus.102224

**Published:** 2026-01-24

**Authors:** Taiki Nishiba, Kazuhisa Yokota

**Affiliations:** 1 Internal Medicine, Mie Prefectural Shima Hospital, Shima, JPN; 2 Infectious Diseases, Tokyo Bay Urayasu Ichikawa Medical Center, Urayasu, JPN

**Keywords:** blood culture, delayed diagnosis, endocarditis, oral hygiene, streptococcus gordonii

## Abstract

Infective endocarditis (IE) in older adults often presents with nonspecific or subtle clinical features, which may delay recognition, especially when antibiotics are initiated before appropriate diagnostic testing. *Streptococcus gordonii* is a common oral organism capable of causing IE. We describe a case of IE caused by levofloxacin-resistant *S. gordonii* in a woman in her 90s with poor oral hygiene. She developed persistent fever after receiving empirical levofloxacin for a presumed urinary tract infection. Although recent dental procedures were absent, blood cultures obtained prior to hospital admission yielded *S. gordonii*. Transthoracic echocardiography revealed severe aortic regurgitation without vegetations, while brain magnetic resonance imaging demonstrated multiple embolic infarctions. The diagnosis of IE was based on the isolation of *S. gordonii* and clinical features consistent with the disease, including fever, severe aortic regurgitation, multiple cerebral embolic infarctions, and glomerulonephritis. The organism was resistant to levofloxacin while remaining susceptible to penicillin and ceftriaxone. Despite administration of appropriate antimicrobial therapy, the patient’s condition deteriorated, and palliative care was pursued in accordance with her wishes. This case underscores two critical diagnostic and management challenges in IE among older adult patients: failure to obtain blood cultures before initiating antibiotics and the potential contribution of poor oral hygiene to bacteremia. To avoid diagnostic delays, it is crucial to obtain blood cultures prior to antibiotic administration. Furthermore, maintaining optimal oral hygiene may help reduce the risk of bacteremia.

## Introduction

Infective endocarditis (IE) remains a life-threatening condition with substantial morbidity and mortality. Its incidence has been rising with an aging population, and the median age of affected individuals continues to increase. Recent registry data indicate that older adult patients with IE frequently have prosthetic valves, cardiac implantable electronic devices, or comorbidities such as diabetes mellitus [1]. These individuals frequently present with atypical or nonspecific symptoms, such as fatigue or appetite loss, rather than the classic febrile presentation, complicating early diagnosis [2]. According to the 2023 European Society of Cardiology (ESC) guidelines, IE should be suspected in patients exhibiting sepsis or fever of unknown origin, even in the absence of a discernible infectious source [3]. Embolic complications, including cerebral infarctions, occur in approximately 25% of presentations and serve as independent predictors of in-hospital mortality, alongside heart failure [4]. Pre-antibiotic blood culture collection is imperative for the diagnosis and management of IE. Prior antibiotic exposure may result in blood culture-negative endocarditis (BCNE), diminishing bacterial detection rates by up to 40%, thereby exacerbating clinical outcomes [5,6].

*Streptococcus gordonii*, a member of the viridans group streptococci (VGS), is a prevalent oral commensal known for its ability to adhere to platelets and cardiac valves through biofilm formation [7]. Among VGS species, *S. gordonii* exhibits one of the highest affinities for cardiac valves and is frequently associated with endocarditis [8]. Recent data suggest that antimicrobial resistance among VGS is increasing, with reduced susceptibility to β-lactams [9]. Poor oral hygiene has been shown to increase the risk of transient bacteremia during routine daily activities such as tooth brushing [10].

We report a case of levofloxacin-resistant *S. gordonii* IE in an older adult woman. This case highlights the diagnostic challenges of IE in older adults, particularly the potential impact of prior antibiotic exposure on microbial detection and subsequent therapeutic decision-making.

## Case presentation

A 92-year-old woman with a history of persistent atrial fibrillation, myocardial infarction, and cerebellar infarction presented to the emergency department with a four-day history of fever (maximum body temperature, 38.1°C). Nine years earlier, she had sustained an acute myocardial infarction, which was managed medically without percutaneous coronary intervention. At that time, transthoracic echocardiography (TTE) revealed mild aortic regurgitation with preserved left ventricular systolic function.

Four days prior to admission, the patient developed a progressive loss of appetite. The following day, rapid antigen tests for influenza and coronavirus disease returned negative. Three days before admission, fever was the patient’s only symptom, and no evaluation, including urinalysis, was performed to identify the source of infection. A urinary tract infection was presumed at her primary care clinic, and levofloxacin was prescribed empirically. Due to persistent fever, referral was made to the hospital for further evaluation. Neither chest pain nor dyspnea was reported, and there was no history of recent dental procedures. Allergy and family history profiles were unremarkable. Regular medications comprised cilostazol, apixaban, lansoprazole, enalapril, carvedilol, and azosemide.

On arrival, the patient was alert but appeared clinically compromised. The vital signs were as follows: temperature was 37.6°C, blood pressure was 100/63 mmHg, heart rate was 115 beats per minute, respiratory rate was 20 breaths per minute, and oxygen saturation was 96% on ambient air. Glasgow Coma Scale score was E3V5M5. Physical examination revealed a grade 2/6 diastolic murmur audible at the aortic area, along with poor oral hygiene characterized by multiple dental caries. No peripheral signs of endocarditis, such as Osler’s nodes or Janeway lesions, were observed.

Laboratory investigations revealed leukocytosis and elevated inflammatory markers. Serum creatinine was elevated, indicating acute kidney injury, and B-type natriuretic peptide was markedly increased. Urinalysis revealed hematuria with red blood cell casts. Detailed laboratory values are summarized in Tables 1, 2.

**Table 1:** Laboratory findings on admission

Test	Result	Reference range
White blood cell count (/µL)	7,700	3,300-8,600
Hemoglobin (g/dL)	12.4	11.6-14.8
Hamatocrit (%)	36.4	35.1-44.4
Platelet (/µL)	193,000	158,000-348,000
Albumin (g/dL)	3.7	4.1-5.1
Total bilirubin (mg/dL)	1.4	0.4-1.5
Aspartate aminotransferase (IU/L)	32	13-30
Alanine aminotransferase (IU/L)	16	7-23
Lactate dehydrogenase (IU/L)	263	124-222
Alkaline phosphatase (IU/L)	79	28-113
Blood urea nitrogen (mg/dL)	116	8-20
Creatinine (mg/dL)	2.93	0.46-0.79
C-reactive protein (mg/dL)	9.5	0.00-0.14
Glucose (mg/dL)	157	73-109
Sodium (mmol/L)	148	138-145
Potassium (mmol/L)	4.1	3.6-4.8
Chloride (mmol/L)	108	101-108
B-type natriuretic peptide (pg/mL)	2142.8	0-18.4

**Table 2:** Urinalysis HPF: high-power field

Test	Result	Reference range
Dipstick analysis		
Protein	±	Negative
Occult blood	2	Negative
Nitrite	Negative	Negative
Leukocyte esterase	2	Negative
Urinary sediment analysis		
Bacteria (/HPF)	None	None
White blood cells (/HPF)	10-19	<4
Red blood cells (/HPF)	30-49	<4
Red blood cell casts	Present	None

**Figure 1:**
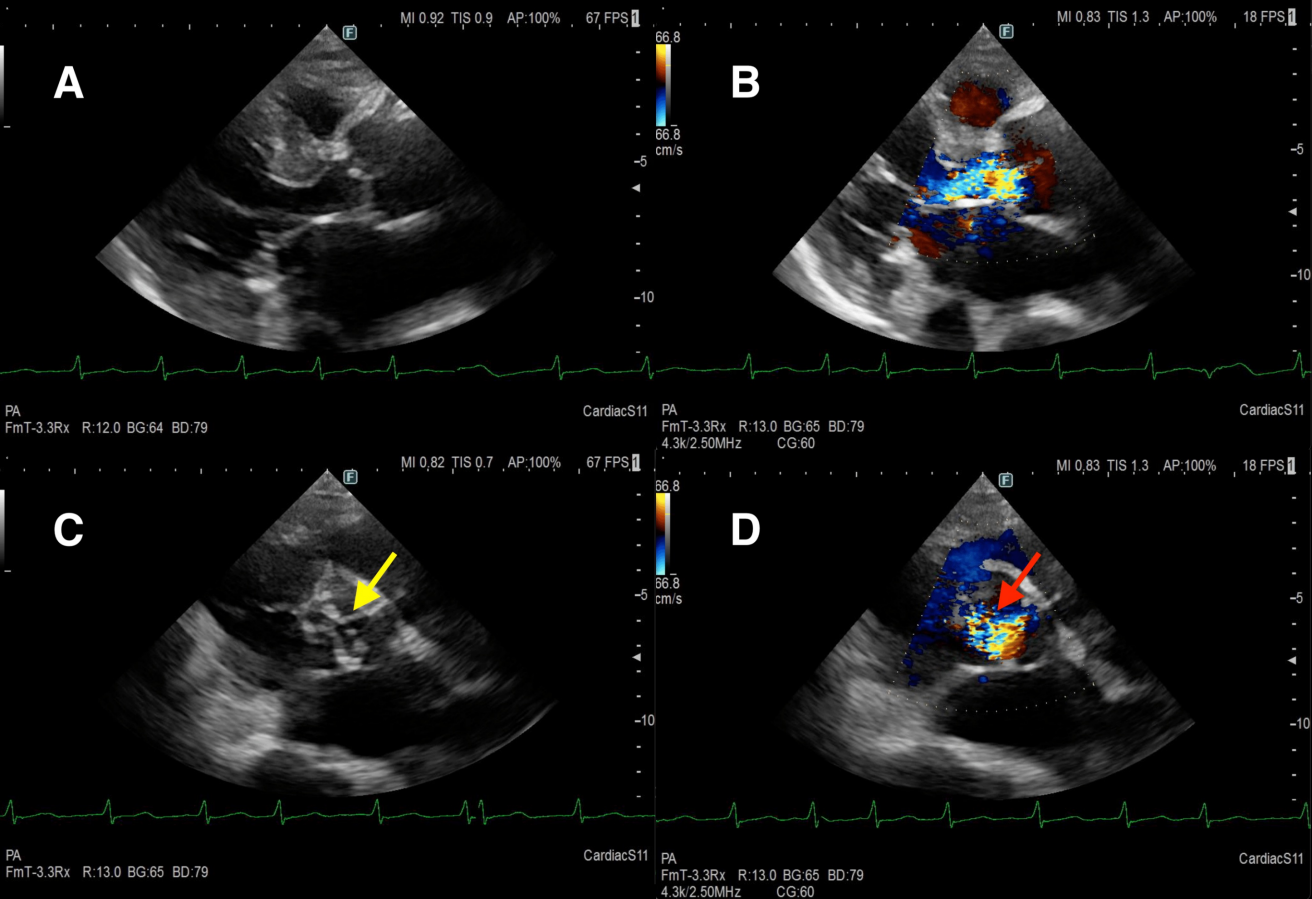
Transthoracic echocardiography Parasternal long‑axis (A, B) and short‑axis (C, D) transthoracic echocardiographic views were obtained on admission day. (A) Two-dimensional parasternal long-axis view shows no obvious valvular vegetations. (B) Color Doppler parasternal long-axis view demonstrates severe aortic regurgitation with an eccentric regurgitant jet. (C) Two-dimensional parasternal short-axis view shows incomplete coaptation of the left coronary cusp (yellow arrow). (D) Color Doppler parasternal short-axis view demonstrates an eccentric regurgitant jet originating near the left coronary cusp commissure (red arrow).

**Figure 2:**
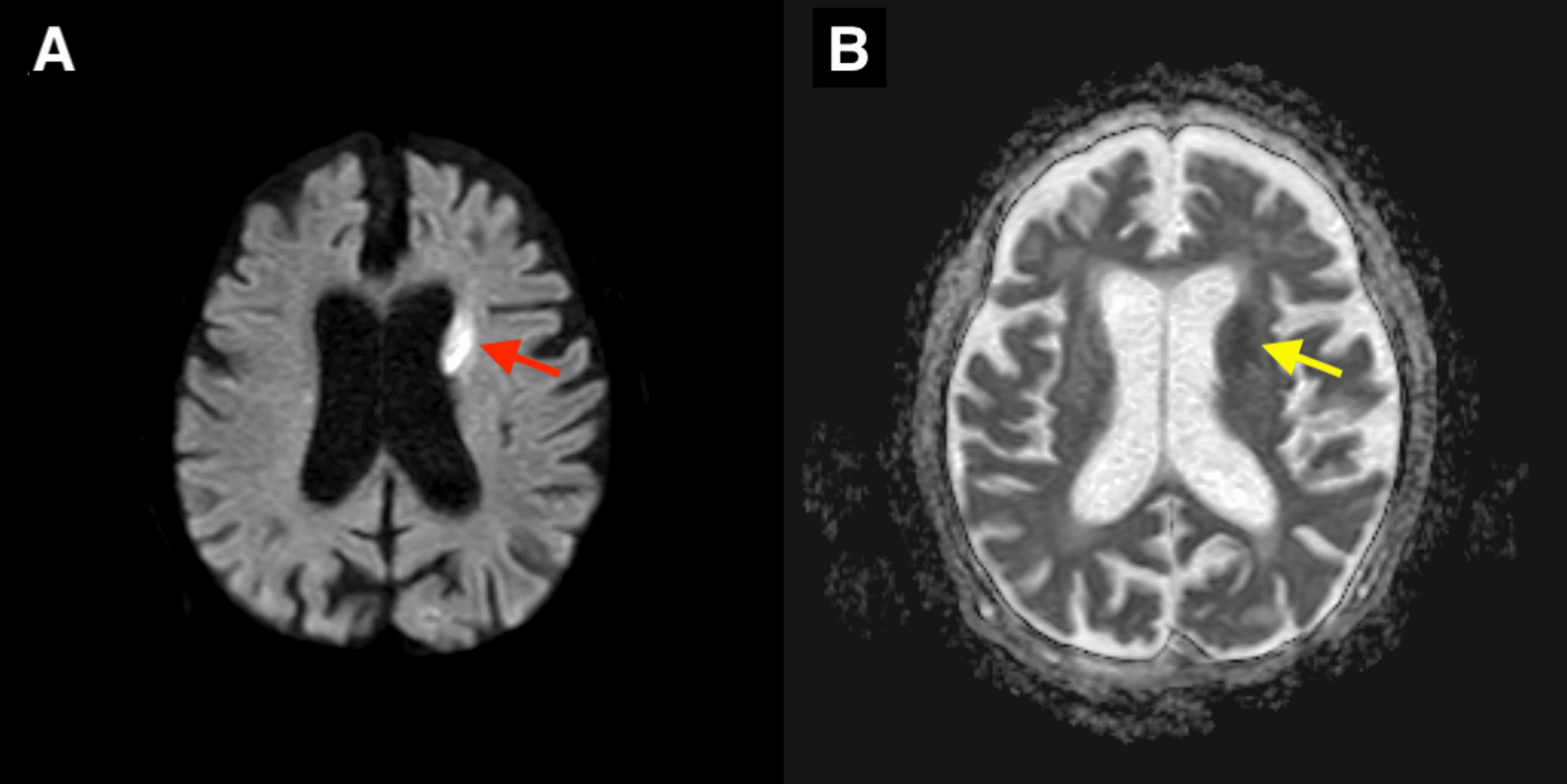
Brain magnetic resonance imaging (MRI) A brain MRI was performed on hospital day 6. Axial diffusion-weighted image (DWI) (A) reveals a hyperintense acute infarct in the left caudate nucleus (red arrow), and the corresponding apparent diffusion coefficient (ADC) map (B) demonstrates a hypointense signal in the same region (yellow arrow).

TTE showed severe aortic regurgitation without visible vegetations. The left coronary cusp exhibited incomplete coaptation, resulting in an eccentric regurgitant jet directed toward the left ventricular outflow tract, suggestive of potential cusp destruction or perforation (Figure 1). TEE was considered to further evaluate for vegetations, but it was not performed because the patient was too frail to tolerate the procedure. Whole-body computed tomography revealed mild cardiomegaly. Brain magnetic resonance imaging demonstrated multiple acute infarcts, including involvement of the caudate nucleus (Figure 2).

**Figure 3:**
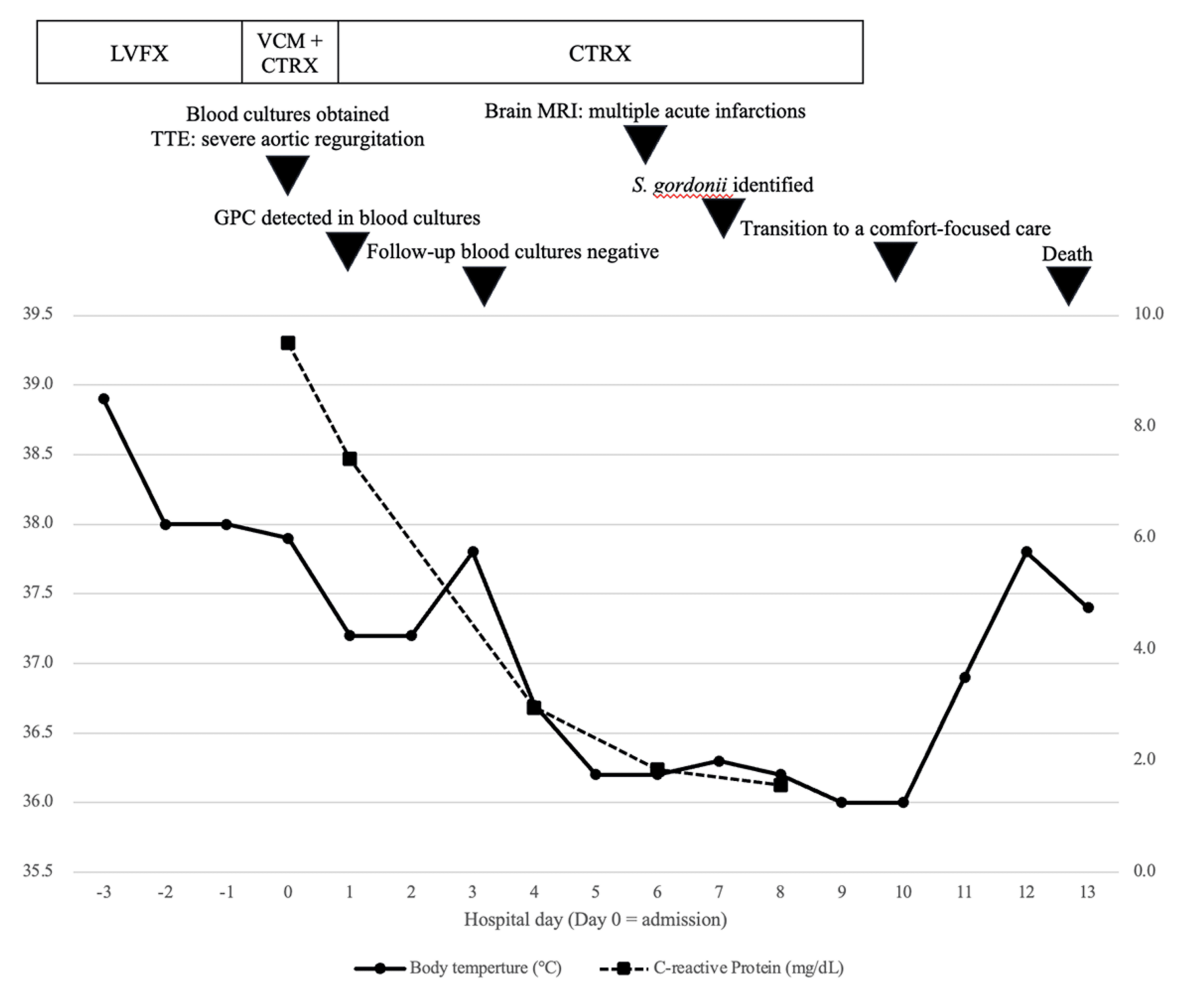
Clinical course and treatment after hospitalization The upper panel depicts antimicrobial therapy and key clinical events. The lower panel shows body temperature (left y-axis) and C-reactive protein levels (right y-axis) by hospital day (day 0=admission). LVFX: levofloxacin; VCM: vancomycin; CTRX: ceftriaxone; TTE: transthoracic echocardiography; MRI: magnetic resonance imaging

On day 0 (the day of admission), two sets of blood cultures were obtained, and the patient was hospitalized. Based on the progression of pre-existing aortic regurgitation, embolic cerebral infarction, and persistent fever, IE was suspected at admission. Empirical antimicrobial therapy with piperacillin-tazobactam and vancomycin was initiated. The subsequent clinical course, including antimicrobial therapy and microbiological findings, is summarized in Figure 3. On the day after admission, gram-positive cocci were reported from both sets of blood cultures; the antimicrobial regimen was changed to ceftriaxone. On day 7, *S. gordonii* was identified using matrix-assisted laser desorption/ionization time-of-flight mass spectrometry with a confidence score of 2.18. Antimicrobial susceptibility testing was performed at an external laboratory and interpreted according to the Clinical and Laboratory Standards Institute (CLSI) M100 (26th edition) [11]. The organism was susceptible to penicillin, ceftriaxone, and vancomycin but resistant to levofloxacin (Table 3). Follow-up blood cultures obtained on hospital day 3 were negative, confirming bacteremia clearance. According to the Duke criteria, the diagnosis of endocarditis was considered definite, with one major criterion (pathogen identified in blood cultures) and four minor criteria (fever, progression of pre-existing aortic regurgitation, intracranial ischemic lesions, and glomerulonephritis).

**Table 3:** Antimicrobial susceptibility profile of Streptococcus gordonii isolated from blood cultures Minimum inhibitory concentrations and corresponding susceptibility interpretations for *Streptococcus gordonii* isolated from blood cultures are shown. Susceptibility interpretations were based on Clinical and Laboratory Standards Institute (CLSI) M100 (26th edition) [11].

Drug	Minimum inhibitory concentration (μg/mL)	Interpretation
Penicillin	≦0.06	Susceptible
Ampicillin	≦0.25	Susceptible
Ceftriaxone	≦0.12	Susceptible
Vancomycin	≦0.5	Susceptible
Levofloxacin	≧8	Resistant

Despite antimicrobial therapy, the patient continued to experience anorexia and poor oral intake, became progressively less responsive, and was unable to communicate effectively. Following a multidisciplinary goals-of-care discussion, the management strategy was transitioned to a comfort-focused approach in accordance with previously expressed patient preferences. Antimicrobial therapy was discontinued because it was unlikely to provide further symptomatic benefit in the palliative setting. The patient died on hospital day 13.

## Discussion

This case highlights two gaps in the diagnosis and management of IE in older adult patients: (1) the initiation of antibiotic therapy prior to obtaining blood cultures and (2) inadequate oral hygiene as a potential source of bacteremia. These factors, combined with the specific microbiological features of *S. gordonii*, contributed to the diagnostic and therapeutic challenges in this case.

Accurate pathogen identification is essential for the diagnosis and management of IE [12]. In this case, *S. gordonii* was successfully isolated; however, the organism exhibited resistance to levofloxacin, the empiric antibiotic prescribed by the primary physician. Paradoxically, this ineffective antibiotic exposure allowed the pathogen to persist in the bloodstream, enabling pathogen detection via blood cultures. If the initial antibiotic had been effective, blood cultures might have turned negative, resulting in BCNE. This illustrates a primary care gap - the failure to obtain pre-treatment blood cultures. Administering empirical antibiotics before culture collection is a major cause of BCNE, reducing culture positivity by approximately 35%-40% and increasing complication and mortality rates [13]. The 2023 ESC guidelines emphasize the importance of obtaining blood cultures prior to initiating therapy, even in older adult or septic patients [3]. The diagnostic gap is primarily attributable to the inherent challenges of recognizing IE in older adults. In this population, IE frequently presents with atypical symptoms such as malaise, anorexia, or low-grade fever rather than the classical febrile presentation [14]. Such nonspecific features frequently result in premature diagnostic closure and empiric treatment for more common conditions like urinary tract infection. Additionally, the 2023 ESC guidelines emphasize that invasive procedures and surgery are often withheld in older adult patients with IE, potentially resulting in diminished diagnostic assertiveness and therapeutic intensity [3]. Clinicians should recognize that this tendency may impede both diagnosis and optimal treatment in this population. Although TEE offers enhanced sensitivity over TTE for detecting vegetations and abscesses, its use is sometimes constrained by concerns about invasiveness [3]. While TEE is not feasible, short-interval repetition of TTE is recommended to improve diagnostic accuracy [3]. In the present case, no vegetation was detected by TTE, and TEE might have been indicated to reinforce diagnostic certainty. However, considering the patient’s frailty, the diagnosis of IE was based on the cumulative findings of progressive aortic regurgitation, embolic infarctions, *S. gordonii* bacteremia, and glomerulonephritis. This case underscores the importance of balancing diagnostic accuracy with the patient’s overall condition and frailty when evaluating suspected IE in older adults.

The second care gap in this case was suboptimal oral hygiene, which likely contributed to bacteremia and subsequent IE. Poor oral hygiene can cause gingival inflammation and mucosal disruption, facilitating translocation of oral bacteria into the bloodstream, even following routine toothbrushing. Individuals with elevated levels of dental plaque or calculus exhibit a markedly increased risk of post-brushing bacteremia [10]. *S. gordonii*, a member of the VGS, is a common oral commensal but also a notable pathogen associated with IE. Registry data indicate that approximately 44% of individuals with *S. gordonii* bacteremia are complicated by IE, with an odds ratio of 80.8 when compared with *S. pneumoniae* [8]. The organism expresses adhesion molecules such as Hsa and PadA, which mediate platelet binding and promote attachment to valvular tissue, facilitating biofilm formation [15]. This property contributes to valvular destruction and embolic complications, as evidenced by the patient’s severe aortic regurgitation and multiple cerebral infarctions. In recent years, rising antimicrobial resistance among *Streptococcus* species, including VGS, has been reported. Nevertheless, β-lactam antibiotics remain the first-line agents for the treatment of VGS endocarditis [3]. For penicillin-susceptible isolates, a four-week course of intravenous penicillin G or ceftriaxone is recommended. In contrast, combination therapy with gentamicin is indicated for isolates exhibiting intermediate resistance. In this case, the isolate remained susceptible to β-lactams, thereby supporting the transition to ceftriaxone. However, fluoroquinolone resistance among *Streptococcus* species has been increasingly documented worldwide, with reported rates ranging from 11.5% to 47.9% [16]. Consequently, fluoroquinolones are not recommended as empiric therapy for suspected IE, particularly when VGS are a potential pathogen. To prevent IE, the 2023 ESC guidelines emphasize maintaining oral hygiene and routine dental care over antibiotic prophylaxis [3]. Close interdisciplinary collaboration between medical and dental professionals is particularly significant for older adults or dependent individuals, as reducing oral bacterial burden is key to mitigating the risk of bacteremia and IE recurrence.

In summary, this case highlights two modifiable care gaps: failure to obtain blood cultures prior to empirical antibiotic initiation and suboptimal oral hygiene. Both are preventable contributors to diagnostic delay and infection risk in older adults. Recognizing these factors, alongside the distinct pathogenicity of *S. gordonii, *can support clinicians in enhancing diagnostic precision, optimizing infection control, and improving clinical outcomes.

## Conclusions

This case underscores the diagnostic challenges of IE caused by levofloxacin-resistant *S. gordonii* in a frail older adult patient. It highlights two preventable care gaps in the diagnosis and management of IE in older adult patients: failure to obtain blood cultures prior to antibiotic administration and inadequate oral hygiene, which may have contributed to bacteremia as a plausible source. Prompt diagnostic evaluation, including blood cultures and echocardiography, is essential to prevent culture-negative IE and facilitate timely treatment. Furthermore, maintaining optimal oral hygiene may help reduce the risk of IE and enhance clinical outcomes in older adults. However, these findings should be interpreted with caution because this is a single case, and TEE was not performed, so detailed valvular involvement could not be confirmed.
